# Sneddon-Wilkinson Disease as a Manifestation of Severe Hypothyroidism

**DOI:** 10.7759/cureus.82908

**Published:** 2025-04-24

**Authors:** Lara I Shehadeh, Ethan Matthew, Michelle Tarbox

**Affiliations:** 1 Department of Dermatology, School of Medicine, Texas Tech University Health Sciences Center, Lubbock, USA; 2 Department of Dermatology, Texas Tech University Health Sciences Center, Lubbock, USA

**Keywords:** dermatologic manifestations, dermatology, hypothyroidism, sneddon-wilkinson disease, swd

## Abstract

A Caucasian female in her 80s with a past history of hypothyroidism, chronic kidney disease, lichen sclerosus, and intertrigo presented originally with a pustular rash to an outpatient dermatology office. The patient was treated for intertrigo with nystatin and oral fluconazole before re-presenting four months later with new erythematous plaques and overlying small pustules in the groin, axilla, and inframammary folds. A shave biopsy was taken, and the patient was treated with triamcinolone for plaques on the abdomen and nystatin and hydrocortisone for intertriginous areas. After two weeks, the patient was admitted to the hospital with expressive aphasia and a presumed stroke, and an EEG consistent with metabolic encephalopathy. Her thyroid-stimulating hormone (TSH) level was found to be 29 mlU/L (reference: 0.27-4.20 mlU/L) and T4 level of 0.42 ng/dL (reference: 0.93-1.70 ng/dL), reflective of severe hypothyroidism. The patient was started on IV levothyroxine and methylprednisolone, with improvement in mental status and rash within six days of admission to the hospital. A review of initial biopsy histology showed subcorneal accumulation of neutrophils with spongiosis, consistent with Sneddon-Wilkinson disease (SWD). SWD commonly manifests as painless superficial pustular lesions arising within erythematous plaques. It is found to often be associated with multiple comorbidities, including thyroid disorders. Given this unique presentation of SWD prior to the onset of severe hypothyroidism and myxedema coma, clinicians should consider examining thyroid hormone levels in such patients to rule out hyperthyroidism and hypothyroidism.

## Introduction

Sneddon-Wilkinson disease (SWD), or classic subcorneal pustular dermatosis, commonly manifests as painless superficial pustular lesions arising within erythematous plaques [1]. Histologically, lesions are represented as subcorneal neutrophilic aggregates, and older lesions may exhibit spongiosis or acantholysis [2]. These cutaneous lesions typically present on the trunk and intertriginous regions and may coalesce to form annular patterns [3]. SWD commonly affects middle-aged or elderly women, between the ages of 50 and 70 years [4,5]. SWD is also found to often be associated with comorbidities, commonly with pyoderma gangrenosum and benign monoclonal IgA gammopathy, but also with rheumatoid arthritis and thyroid disorders [3,4]. This case report cites the first case of SWD occurring concurrently with myxedema coma.

## Case presentation

**Figure 1:**
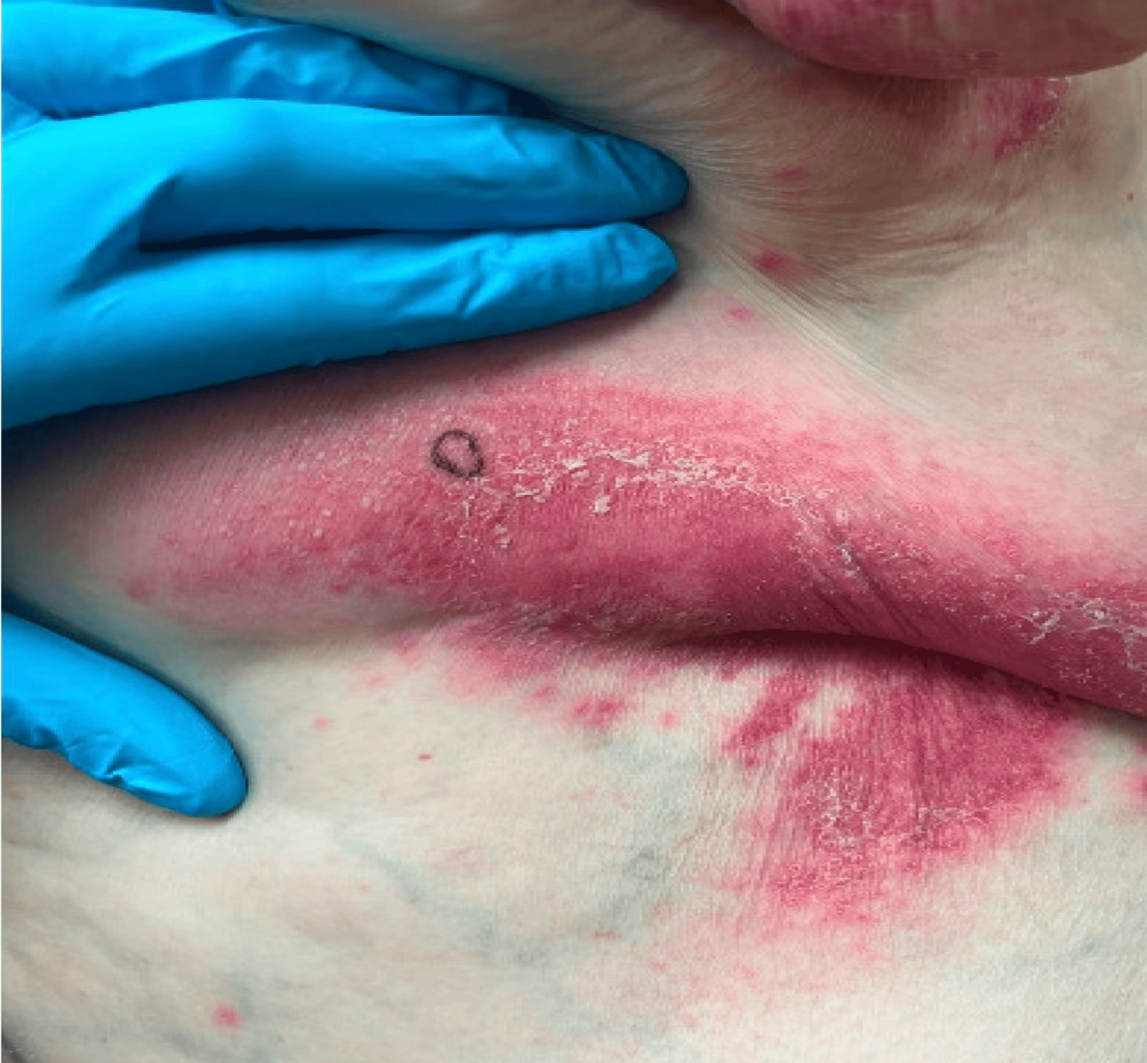
Cutaneous involvement of the intertriginous region on the trunk.

**Figure 2:**
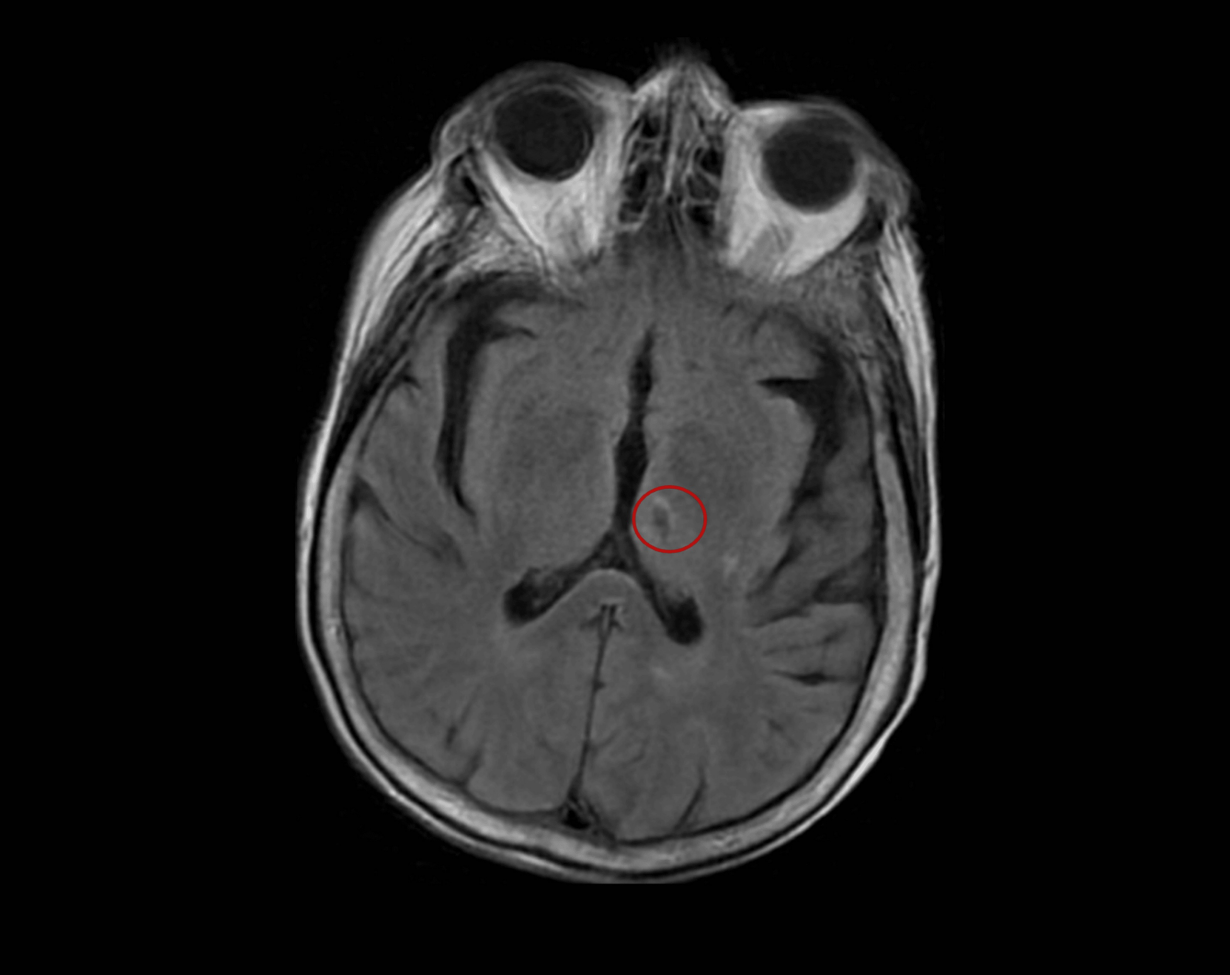
MRI of lacunar stroke.

An 88-year-old Caucasian female with a past medical history of hypothyroidism, chronic kidney disease, lichen sclerosus, and intertrigo presented with a pustular rash to an outpatient dermatology office. She was previously treated for intertrigo of the axilla and groin, with minimal response to nystatin and oral fluconazole. She represented four months later with minimal improvement and new findings of pustules overlying erythematous plaques (Figure 1). A biopsy was taken, and the patient was treated with triamcinolone for plaques on the abdomen and nystatin and hydrocortisone for intertriginous areas. Two weeks later, the patient was admitted to the hospital after presenting to the ER with expressive aphasia, slowed speech, and nonpitting edema. The patient underwent workup for acute stroke. Computed tomography angiography showed no acute intracranial pathology, including ischemic or hemorrhagic stroke. MRI of the brain revealed an old lacunar stroke and changes consistent with chronic small vessel disease (Figure 2). EEG demonstrated generalized slowing, consistent with metabolic encephalopathy.

Initial laboratory values were consistent with hypothyroidism, with a thyroid-stimulating hormone (TSH) level of 29 mlU/L (normal: 0.27-4.20 mlU/L) and T4 of 0.42 ng/dL (normal: 0.93-1.70 ng/dL). Given her altered mental status on admission, the patient was treated for myxedema coma and started on IV levothyroxine 50 mcg daily. Labs showed her neutrophil count increased from 15.44 to 31.94 x 10^9^/L (normal: 2.0-7.5 x 10^9^/L), and hemoglobin was 11 mg/dL (normal: 12-16 mg/dL). Potassium was elevated at 6.4 mEq/L (normal: 3.5-5.5 mEq/L) secondary to her chronic kidney disease. The patient was initially treated with methylprednisolone for suspicion of a drug reaction prior to being seen by dermatology.

**Figure 3:**
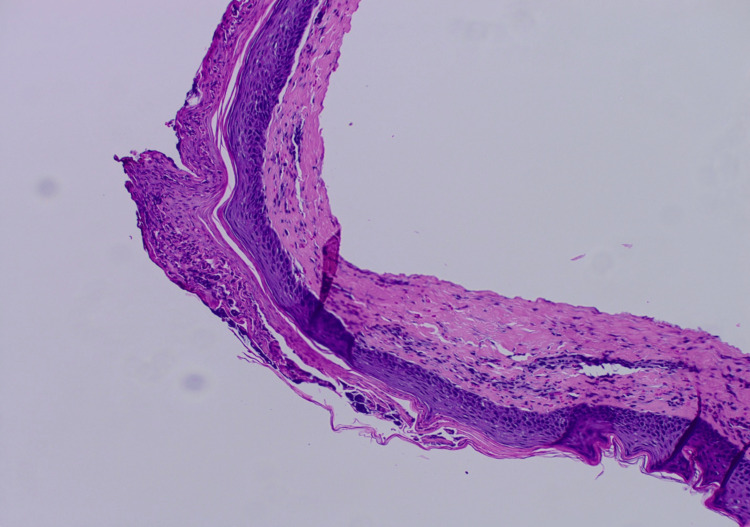
Subcorneal neutrophil accumulation and epidermal spongiosis. Hematoxylin and eosin (H&E) stain, original magnification x100.

**Figure 4:**
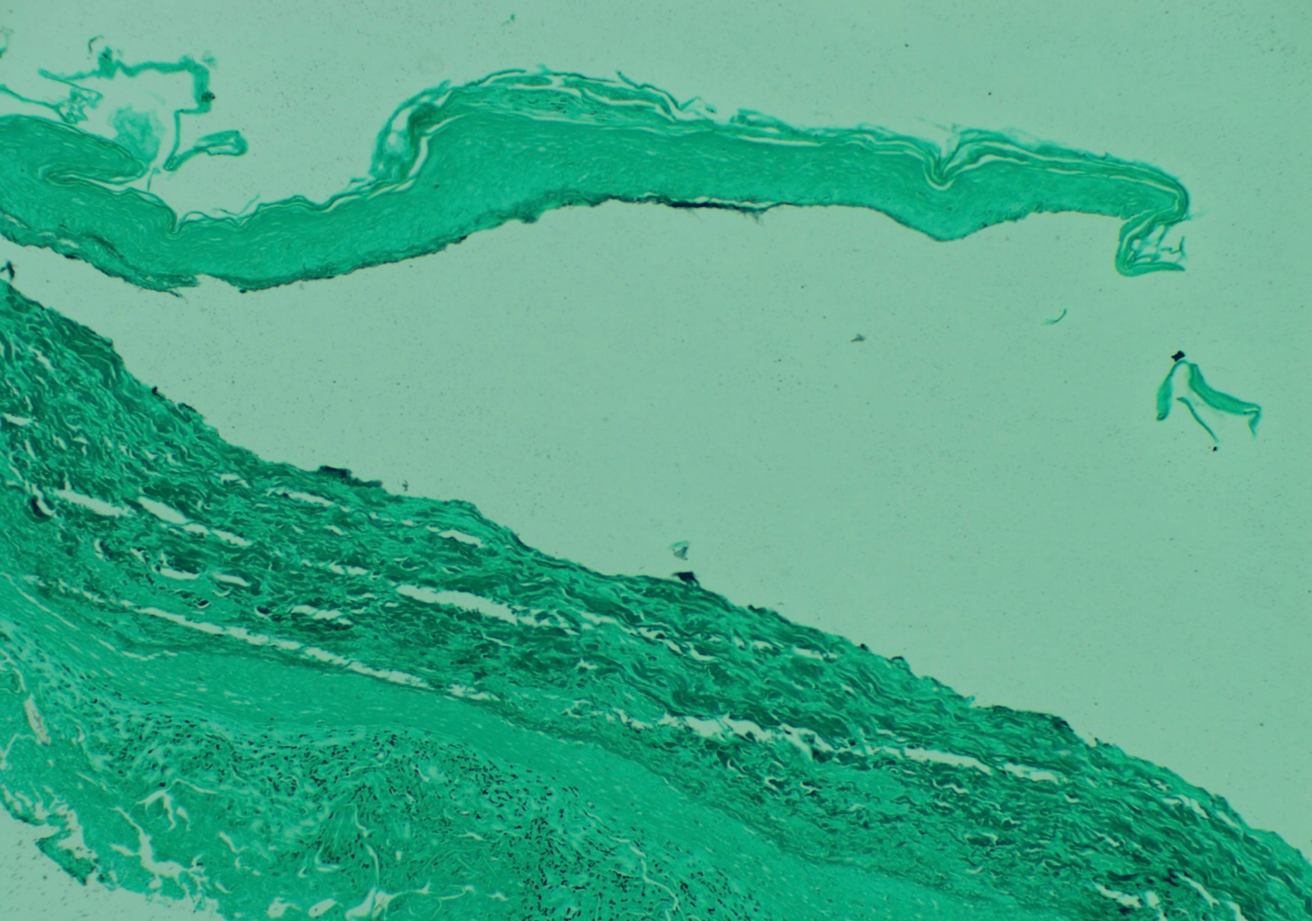
Grocott's methenamine silver (GMS) stain was negative for fungus.

For the first three days of admission, encephalopathy had minimal improvement, until the fourth day of admission when the patient’s mentation improved with thyroid replacement. Dermatology saw the patient on day five of her admission, at which time the pustular aspect of the rash had improved with methylprednisolone, topical triamcinolone, and IV levothyroxine (50 mg). Upon assessment by dermatology, differential diagnosis included pustular psoriasis, SWD, and IgA pemphigus. Review of initial biopsy histology was consistent with SWD, showing subcorneal accumulation of neutrophils with spongiosis (Figure 3). Grocott's methenamine silver (GMS) stain was negative, ruling out fungal infection (Figure 4). Dermatology recommended continuation of hydrocortisone, triamcinolone, and thyroid replacement for treatment of SWD, and nystatin cream for intertrigo prevention. On the sixth day of admission, mentation improved significantly, and she became more alert and oriented, with significant improvement of the rash. She was discharged home, and the patient was lost to follow-up, with no return to the clinic.

## Discussion

Though SWD has been associated with hyperthyroidism in the literature, to the authors' knowledge, there are no reports associated with SWD as a heralding symptom of myxedema coma. This represents the first case of SWD as a presenting symptom prior to myxedema coma. In a previous case series, hypothyroidism and hyperthyroidism were detected in patients with SWD; however, the case series did not expand on the role of thyroid levels in the etiology of SWD [6]. One case also found there to be an association of SWD with hyperthyroidism [7]. Proposed mechanisms of SWD pathogenesis include neutrophil migration to the epidermis in response to elevations in chemokines, including tumor necrosis factor-alpha (TNFα), interleukin-8, and C5a. Elevations of these chemokines are seen in patients with SWD [8]. In a study performed by Tayde et al., they note that the levels of TNFα were elevated within hypothyroid patients compared to healthy controls [9]. Though this is one study, TNFα elevation in patients with hypothyroidism may be a driver in the development of SWD within this patient group.

These findings suggest that evaluation of thyroid levels may be essential in the workup and initial examination of SWD. In this presented case, routine screening of thyroid hormone may have identified her underlying endocrinopathy and led to the treatment of her severe hypothyroidism prior to her presentation of myxedema coma and admission to the hospital. In this patient, lesions of SWD began to clear with the administration of IV levothyroxine while admitted. Unfortunately, the patient was lost before follow-up could occur, and complete clearance of lesions could not be confirmed.

The pathophysiology of SWD is still unclear, but previous studies have evaluated the most common associations of SWD being pyoderma gangrenosum, benign monoclonal IgA gammopathy, and multiple myeloma [4,5]. SWD has also been found to be associated with autoimmune disorders, including systemic lupus erythematosus (SLE), Sjögren’s syndrome, rheumatoid arthritis, and multiple sclerosis [4]. Treatment of SWD includes topical steroids and immunosuppressants. Successful improvement of symptoms for SWD was seen with the use of oral dapsone daily (50-200 mg) and may be first line in recalcitrant cases [10]. Recurrence of SWD has been common for patients who ceased treatment, so maintenance therapy at a lower dosage may be needed for suppression [10]. Topical or oral steroids can also be used as a standalone treatment or alongside oral dapsone [3]. This combination of treatment produces the best result for the treatment of SWD. Additionally, previous cases have demonstrated that the use of ultraviolet light therapy [10], anti-tumor necrosis factor therapy [10], and infliximab [11] also yields treatment responses.

## Conclusions

Given this unique presentation of SWD prior to the onset of severe hypothyroidism and myxedema coma, it is the recommendation of the authors that patients undergo examination of the thyroid to rule out endocrinopathy. Laboratory evaluation of the thyroid and appropriate replacement with IV levothyroxine were instrumental in diagnosing and eventually improving both the symptoms of myxedema coma and SWD in this patient. Given the association of thyroid abnormalities in previous case series and the presentation of SWD preceding myxedema coma in this patient, the association of thyroid abnormalities as a possible etiology should be explored.

## References

[1] Bolognia JL, Schaffer JV, Cerroni L (2017). Dermatology.

[2] Bhargava S, Kumar U, Kroumpouzos G (2020). Subcorneal pustular dermatosis: comprehensive review and report of a case presenting during pregnancy. Int J Womens Dermatol.

[3] Mayba J, Hawkins CN (2019). First presentation of Sneddon-Wilkinson disease with unexpected immunoglobulin A gammopathy: a case report and review of the literature. SAGE Open Med Case Rep.

[4] Manjaly P, Sanchez K, Gregoire S, Ly S, Kamal K, Mostaghimi A (2024). Superficial and bullous neutrophilic dermatoses: Sneddon-Wilkinson, IgA pemphigus, and bullous lupus. Dermatol Clin.

[5] Cheng S, Edmonds E, Ben-Gashir M, Yu RC (2008). Subcorneal pustular dermatosis: 50 years on. Clin Exp Dermatol.

[6] Narayanan SV, Anitha K (2020). Clinical and histopathological profile of subcorneal pustular dermatosis. J Evolution Med Dent Sci.

[7] Taniguchi S, Tsuruta D, Kutsuna H, Hamada T (1995). Subcorneal pustular dermatosis in a patient with hyperthyroidism. Dermatology.

[8] Tajiri K, Nakajima T, Kawai K, Minemura M, Sugiyama T (2015). Sneddon-Wilkinson disease induced by sorafenib in a patient with advanced hepatocellular carcinoma. Intern Med.

[9] Tayde PS, Bhagwat NM, Sharma P (2017). Hypothyroidism and depression: are cytokines the link?. Indian J Endocrinol Metab.

[10] Watts PJ, Khachemoune A (2016). Subcorneal pustular dermatosis: a review of 30 years of progress. Am J Clin Dermatol.

[11] Bonifati C, Trento E, Cordiali Fei P, Muscardin L, Amantea A, Carducci M (2005). Early but not lasting improvement of recalcitrant subcorneal pustular dermatosis (Sneddon-Wilkinson disease) after infliximab therapy: relationships with variations in cytokine levels in suction blister fluids. Clin Exp Dermatol.

